#  Congenital Hernia of the Umbilical Cord associated with type IIIa Ileal Atresia

**DOI:** 10.21699/jns.v5i4.433

**Published:** 2016-10-10

**Authors:** Rahul Gupta, Praveen Mathur, Pradeep Kumar Gupta

**Affiliations:** Department of Paediatric Surgery, SMS Medical College; Jaipur 302004, Rajasthan, India

**Dear Sir**

Congenital hernia of the umbilical cord is rare; only few cases of its association with other congenital intestinal anomalies have been previously described in the literature. [1-5] We report a case of congenital hernia of the umbilical cord associated with type IIIa ileal atresia in a newborn. 

A 2-day-old, term male neonate, weighing 2.1 kg, 1st in birth order was born by normal delivery. He presented to our department with a small fleshy swelling at the base of the umbilicus, bilious vomiting and epigastric fullness. Antenatal ultrasounds were not done. On examination, the baby was hemodynamically stable, pulse rate-150/min and respiratory rate-50/min. anicteric and mildly dehydrated. Abdominal examination revealed hernia of umbilical cord. There was mild epigastric distension, soft on palpation and absent bowel sounds; nasogastric aspirate was bilious. Laboratory tests were normal except for hypocalcaemia (serum calcium-8 mg/dl) and raised C-reactive protein. Upright abdominal radiograph showed dilated small bowel loops with multiple air-fluid levels with paucity of distal air; there were multiple defects involving the lumbar vertebra (Fig.1). Laparotomy revealed proximal ileal atresia, type IIIa. The proximal part of distal unused ileum was herniating into the umbilical cord, ending blindly and stuck inside the sac of hernia of umbilical cord (Fig.1). It was dissected and freed; end to back ileoileal anastomosis was performed after excision of 10-12cms of dilated loop and confirming distal bowel patency. Post-operative recovery was uneventful and the child is doing well at follow-up.

**Figure:**
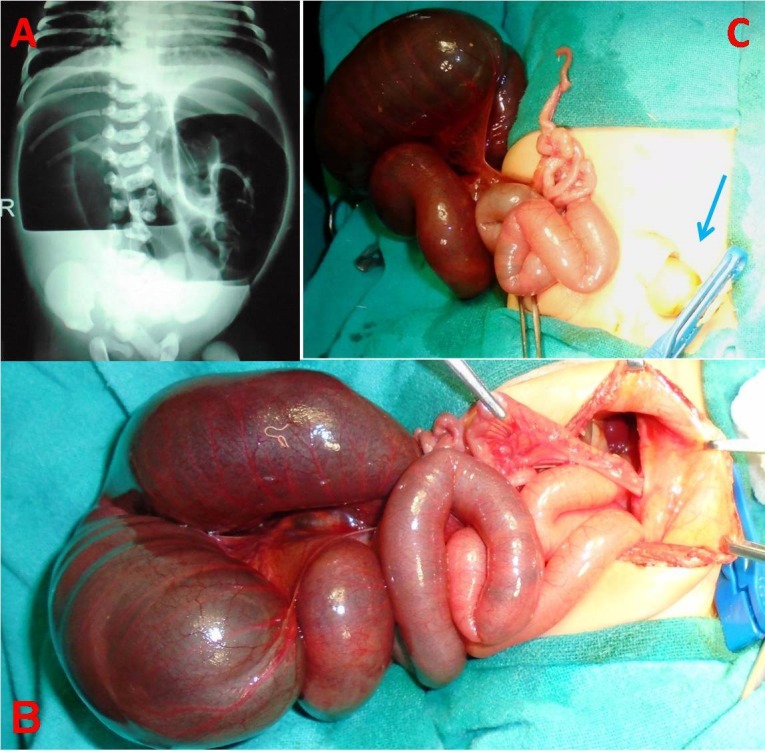
Figure 1: Upright abdominal radiograph showing dilated small bowel loops, multiple air-fluid levels with absence of air distally (A); Intra-operative picture showing type IIIa ileal atresia with distal unused ileum is seen herniating into the umbilical cord (B); hernia of umbilical cord (blue arrow) is seen along with proximal part of distal ileum after its dissection from inside the sac (C).

Hernia of umbilical cord has a benign appearance, surgical correction is easy, and if left as such spontaneous epithelialization of the coverings of the hernia occurs. Hernia of umbilical cord is rarely associated with other congenital anomalies, as against abdominal wall defects (gastroschisis and omphalocele) which are commonly associated. [6] A small number of cases of hernia of umbilical cord associated with intestinal atresia, patent vitellointestinal duct, Meckel's diverticulum, incarceration of liver and gall bladder, and persistent cloaca have been reported earlier. [1-7] Intestinal atresia associated with congenital hernia of umbilical cord is extremely rare. The earlier reported cases were type I proximal jejunal atresia, type IIIb ileal atresia, type I colonic atresia, and jejunal with colonic atresia (congenital short bowel). [1-5] Intestinal atresia occurs as a consequence of in-utero vascular insult. We hypothesize that incarceration of bowel followed by vascular insult due to persistence of herniation of the midgut is the cause of type IIIa ileal atresia in our case. This early vascular accident was followed by resorption of devascularised fetal intestines. The mesenteric defect at the level of ileum and incarceration at umbilical cord and vascular insufficiency leading to entry/exit level intestinal atresia as proposed by Mirza et al appears to be the best possible explanation. [1] 

To summarize, congenital hernia of the umbilical cord presents with fleshy swelling at the base of cord. Its rare association (intestinal anomalies) like intestinal atresias should be borne in mind to achieve better clinical outcome. The vascular insult due to incarceration inside the sac of hernia of umbilical cord is the cause of type IIIa ileal atresia in our case.

## Footnotes

**Source of Support:** Nil

**Conflict of Interest:** None

## References

[1] Mirza B, Mirza A, Hashim I, Saleem M. Hernia of umbilical cord: report of three unusual cases. J Neonat Surg. 2015;4:16. PMC4447469 26034710

[2] Mirza B, Saleem M. Hernia of umbilical cord with congenital short gut. J Neonat Surg. 2014; 3:26. PMC4420328 26023497

[3] Pal K. Congenital hernia of the umbilical cord associated with extracelomic colonic atresia and perforation of gut in a newborn. Afr J Paediatr Surg. 2014; 11:74-6. 10.4103/0189-6725.129241 24647301

[4] Pal K, Nofal A. Umbilical hernia associated with extracelomic intestinal atresia and perforation of the ileum in a newborn. Ann Saudi Med. 2007; 27:212-3. 10.5144/0256-4947.2007.212 PMC6077069 17568164

[5] Hasaniya NW, Premaratne S, Varnes PM, Shin D, Shim W. Hernia into the umbilical cord with incarceration of liver and gall bladder in a newborn. J Pediatr Surg Case Rep. 2013; 1:432-3.

[6] Burns CW, Ogryzlo MA. Congenital hernia into the umbilical cord; two cases, one associated with persistent cloaca. Can Med Assoc J. 1938; 39:438-41. PMC536812 20321146

[7] Soni V, Valse PD, Vyas S. Colonic atresia due to internal herniation through the falciform ligament defect: a case report. J Neonat Surg. 2014; 3:21. PMC4420329 26023492

